# The Education of Mothers of Families

**Published:** 1842-10

**Authors:** 


ART. II.—*The Education of Mothers of Families; or, the Civilization of the Human Race, by Women.* By M. AIMÉ-MARTIN. Translated by EDWIN LEE, Esq.—*London,* 1842. 8vo, pp. 384.

WE not long since perused Dr. Cerise's work, entitled "Des Fonctions et des Maladies Nerveuses dans leurs rapports avec l'Education Sociale et Privée, Morale et Physique," a work to which Mr. Lee, in his appendix to the above translation, makes frequent reference; and although,—from the general nature of its disquisitions, we did not think proper, at the time, to notice it in our pages; yet as a highly useful and scientific ethical treatise, and as embodying many philosophical and important views of education, we were prepared to recommend it to all persons interesting themselves in the subject of which it professed to treat. The same reasons which prevented us from noticing Dr. Cerise's work, applies to the one before us, which, however, is an extremely interesting and valuable production; being that to which the prize of the French Academy was assigned.

The object of the work, as its title implies, is to show that it is through MOTHERS that society is to be regenerated. Seeing the influence which the maternal, more than the paternal, example and precept exercise over children, as proved in the lives of many celebrated persons, it is proposed to improve the education of mothers or women, in order to ensure a corresponding improvement in that of the rising race. Such views are not new, but were insisted on by various moralists of ancient Greece and Rome. And, although we fear that no education, whether applied to mothers or children, will radically remove all the ills and errors of this world, yet we cannot but strongly recommend a scheme so innocent and plausible as this, to the consideration and countenance of every philanthropist.

The translation seems well executed; the work forms pleasing reading; and Mr. Lee has appended to it a useful and sensible treatise on "the prevailing methods of education, and their influence on health and happiness." We recommend the volume as at once instructive and entertaining.

